# Nondestructive lingual variant of isolated mucosal leishmaniasis in an endemic zone: A case report from Nepal

**DOI:** 10.1016/j.jdcr.2025.10.043

**Published:** 2025-10-30

**Authors:** Deepika Neupane, Deeptara Pathak Thapa, Mohan Bhusal, Sandesh Shah, Joshana Shrestha

**Affiliations:** a Consultant Dermatologist, Department of Dermatology, Nepal Medical College and Teaching Hospital, Kathmandu, Nepal; b Professor, Department of Dermatology, Nepal Medical College and Teaching Hospital, Kathmandu, Nepal; c Resident Doctor, Department of Dermatology, Nepal Medical College and Teaching Hospital, Kathmandu, Nepal

**Keywords:** Leishmania donovani, mucosal leishmaniasis, Nepal, nondestructive variant

## Introduction

Visceral leishmaniasis (VL), also known as kala-azar, is a systemic illness primarily attributed to *Leishmania donovani* (*L donovani*) in the Indian subcontinent, commonly presenting with fever, hepatosplenomegaly, and pancytopenia.^1^ Post-kala-azar dermal leishmaniasis (PKDL) is a dermatological manifestation that appears after apparent clinical cure from VL, typically presenting as hypopigmented macules, papules, or nodules primarily on the face, upper limbs, and trunk.^1^^,^^2^

Mucosal involvement in PKDL is rare and usually accompanies widespread cutaneous or systemic disease.^3^ Isolated mucosal presentation, especially affecting the oral cavity, is exceptionally uncommon.^4^ Lesions may involve the lips, buccal mucosa, tongue, and soft palate, often presenting as granulomatous nodules.^4^^,^^5^

We present a rare instance of a nondestructive lingual variant of isolated mucosal leishmaniasis in an immunocompetent woman from an endemic area of Nepal, without systemic symptoms or a prior VL diagnosis. This case underscores the importance of considering leishmaniasis in the differential diagnosis of unusual oral lesions in endemic areas and highlights diagnostic and therapeutic challenges.

## Case report

A 55-year-old female from the Mugu, a VL-endemic district in western Nepal, presented to our outpatient department with a 2-year history of multiple, progressively enlarging, painless nodular lesions localized exclusively to her tongue. The patient reported mild symptoms, primarily slight difficulty swallowing (dysphagia), but denied any constitutional symptoms such as prolonged fever, night sweats, or unintentional weight loss. She had no documented history or clinical signs suggestive of prior VL.

**Fig 1:**
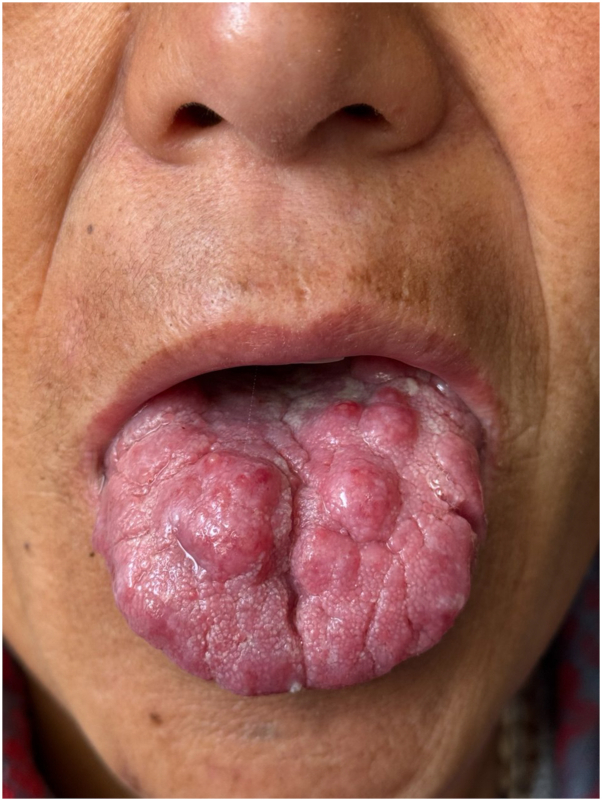
Multiple well-circumscribed, erythematous, round-to-oval nodules distributed across the dorsal and lateral surfaces of the tongue.

On clinical examination, the oral cavity revealed multiple well-circumscribed, erythematous, round-to-oval nodules, approximately 1 to 2 cm in diameter, distributed across the dorsal and lateral surfaces of the tongue (Fig 1). The nodules had a smooth surface texture without evidence of ulceration, bleeding, or secondary infection. A comprehensive systemic examination showed no hepatosplenomegaly, lymphadenopathy, or cutaneous lesions elsewhere on the body, making this an unusual and nondestructive lingual variant of isolated mucosal leishmaniasis.

Given the rarity of exclusive mucosal involvement, the initial differential diagnosis was broad and included infectious diseases such as Hansen’s disease and tuberculosis, along with mucosal neuromas, sarcoidosis, amyloidosis, and mycosis fungoides.^6^, ^7^, ^8^

**Fig 2:**
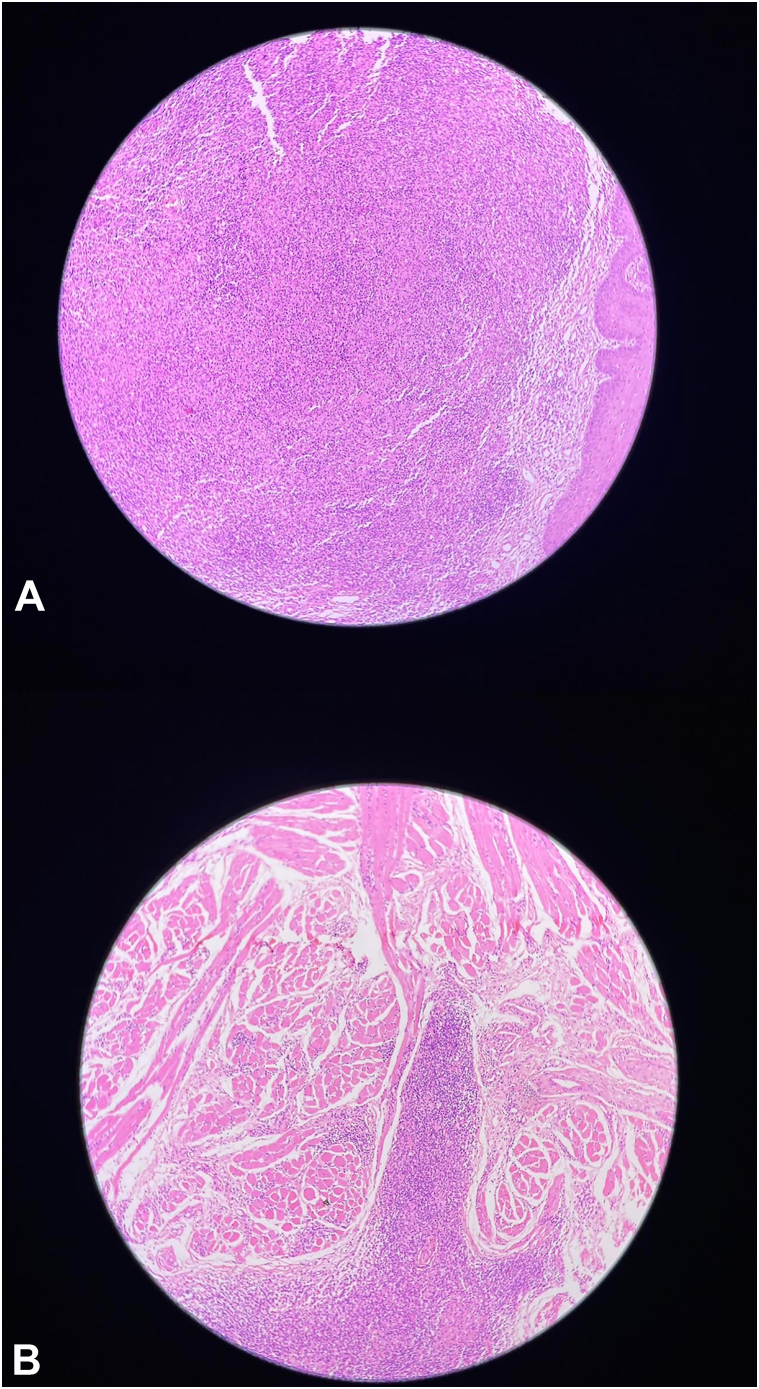
**A,** Hematoxylin and eosin stain, 10×, showing dense dermal infiltrate overlying a fibrotic stroma. **B,** Hematoxylin and eosin stain, 40×, showing inflammatory infiltrate involving striated muscle.

Histopathological examination of an incisional biopsy from one of the tongue lesions revealed hyperplastic stratified squamous epithelium overlying a dense dermal infiltrate and fibrotic stroma interspersed with striated muscle fibers (Fig 2). The submucosa exhibited a dense inflammatory infiltrate composed of lymphocytes, plasma cells, and histiocytes, with multiple well-formed epithelioid granulomas. The inflammatory infiltrate involved the striated muscle as well (Fig 2). No Leishman-Donovan bodies were observed on either hematoxylin and eosin or Giemsa sections despite careful search.

A slit smear from the lingual lesion was also attempted for Giemsa staining; however, due to the tongue’s rich vascularity, sufficient aspirate could not be obtained. Therefore, tissue biopsy was prioritized for histopathological examination.

Despite the absence of a prior VL diagnosis, leishmaniasis was considered based on endemicity and histopathological findings. Although rK-39 serology was positive, its limited sensitivity in mucosal PKDL necessitated molecular confirmation. Polymerase chain reaction (PCR) targeting *L donovani* DNA was performed and returned positive, establishing the diagnosis of isolated lingual PKDL. Slit-skin smear and special stains were negative for acid-fast bacilli and Congo red. Given the association of PKDL with immunosuppression, HIV serology was performed, and it was negative. No histological or clinical features were present to suggest mycosis fungoides.

**Fig 3:**
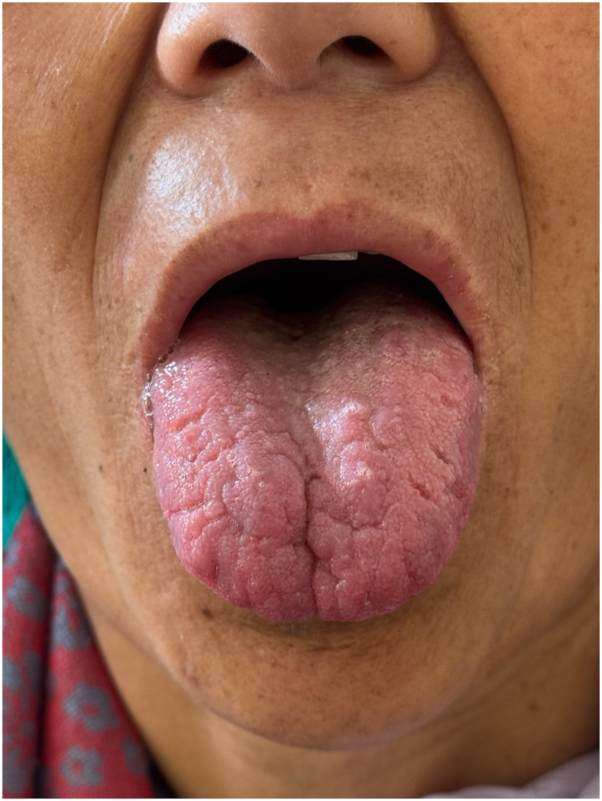
Complete resolution of lingual lesions post-treatment with liposomal amphotericin B.

The patient was started on oral miltefosine (50 mg twice daily), which resulted in early signs of clinical improvement, with partial regression of the nodules. However, after 2 weeks, she developed significant gastrointestinal side effects, including severe abdominal pain and significant weight loss, prompting a dose reduction to 50 mg once daily. Despite this adjustment, her gastrointestinal symptoms persisted, and clinical improvement plateaued. Consequently, therapy was switched to intravenous liposomal amphotericin B (LAMB). The cumulative dose of LAMB was 30 mg/kg, which was administered twice weekly at 5 mg/kg per dose. The patient tolerated LAMB well and achieved complete resolution of the lingual lesions by the end of treatment (Fig 3).

Given the unusual presentation and known risk of relapse in mucosal leishmaniasis, the patient was enrolled in a long-term follow-up program, with scheduled clinical evaluations every 6 months. As of her most recent follow-up at 3 months post-treatment, there has been no evidence of recurrence.

## Discussion

PKDL is a recognized sequelae of VL, typically presenting with macular, papular, or nodular lesions, predominantly on the face, chest, or limbs.^1^^,^^2^ However, isolated mucosal PKDL is exceptionally rare, particularly in the absence of cutaneous or systemic involvement.^3^^,^^4^ In this case, the patient presented with chronic, nonulcerative lingual nodules without a prior VL history—an atypical and diagnostically challenging scenario. Although VL is endemic to parts of Nepal, including Mugu, mucosal variants remain rare and under-reported.^3^

One important differential is mucocutaneous leishmaniasis (MCL), caused by *L donovani*, which has been reported in India, Sri Lanka, and Malta.^5^ Our patient initially presented with papulonodular lesions over the perioral/muzzle area, which regressed spontaneously, while the tongue lesions persisted without ulceration or destruction. Such morphology is more consistent with nondestructive mucosal leishmaniasis, whereas MCL typically presents with destructive or ulcerative lesions.^2^^,^^4^ Similar nondestructive mucosal forms have also been reported in Sudan, where parasites of the *L donovani* complex differed genotypically from classical visceral isolates and in India and Sri Lanka.^9^, ^10^, ^11^ These cases suggest that nondestructive mucosal leishmaniasis may be more geographically widespread than previously recognized, and our case may represent a similar extension into Nepal.

Recent reports from Nepal have also described MCL due to *L donovani*.^12^ However, PKDL remains an important differential in the Indian subcontinent, including Nepal, and can occasionally occur in patients without a documented history of treated VL.^3^^,^^4^ Our consideration of PKDL was based on clinical distribution, epidemiology, and PCR confirmation.^8^^,^^13^^,^^14^ Nonetheless, the absence of prior visceral disease raises the possibility of primary mucosal leishmaniasis in an immunocompetent host, as described in India and Sudan.^10^^,^^11^ To reconcile these overlapping entities, we classify this case as a nondestructive lingual variant of isolated mucosal leishmaniasis.

Differentials included mucosal neuromas, sarcoidosis, mycosis fungoides, amyloidosis, and infections such as tuberculosis and Hansen’s disease.^5^^,^^6^^,^^15^ The presence of linear epithelioid granulomas on histology initially raised suspicion for Hansen’s disease, a common endemic condition, which was carefully excluded based on negative slit-skin smears and histopathological features.^6^ Mucosal neuromas typically present as smooth nodules of the tongue and may be associated with multiple endocrine neoplasia type 2B.^5^ Sarcoidosis and tuberculosis, which may present with similar granulomatous mucosal lesions, were excluded based on histopathological examination and relevant investigations.^15^ Amyloidosis, especially endemic in regions like Mugu, can present with nodular lesions and macroglossia, but was excluded by negative Congo red staining and absence of amyloid deposits on histopathology.^15^ Mycosis fungoides, although rare in the oral cavity, is another important differential and was excluded based on the absence of epidermotropism, Pautrier microabscesses, atypical lymphocytic infiltrates, and lack of systemic involvement.

Histopathology in PKDL is often nonspecific and may mimic granulomatous disorders.^15^ The absence of visible amastigotes is common in chronic mucosal involvement due to a low parasitic burden.^8^ This necessitates molecular diagnostics, with PCR targeting *L donovani* DNA being the gold standard for confirming atypical PKDL.^8^^,^^13^^,^^14^ While rK-39 serological testing is the frontline for VL diagnosis, its sensitivity is reduced in PKDL, especially in mucosal or atypical presentations due to lower antigenic stimulation and antibody titers.^7^^,^^8^^,^^14^ In our patient, a positive rK-39 test supported the diagnosis epidemiologically, but PCR was essential for confirmation.^8^^,^^13^^,^^14^ Although sequencing of the cytochrome oxidase II gene would have clarified whether this isolate shares homology with Sudanese strains, this was not feasible due to resource limitations.^9^

Miltefosine is a first-line agent for PKDL; however, its gastrointestinal toxicity may limit prolonged use.^13^ In such cases, LAMB is a highly effective alternative with a favorable safety profile, rapid action, and good tissue penetration.^13^ Our patient tolerated LAMB well, achieving complete resolution without adverse effects.

The nondestructive clinical course in our patient can be explained by immune mechanisms described in similar cases: a low parasite burden maintained by host defenses, with persistent antigen driving localized immune activation. This limits full clearance but prevents the destructive inflammatory reactions typical of classical MCL.^16^

The prognosis of mucosal leishmaniasis is generally favorable with appropriate treatment; however, recurrence is possible, particularly in atypical or nondestructive variants. Long-term follow-up is therefore essential.

## Conclusion

This case demonstrates an unusual nondestructive lingual variant of isolated mucosal leishmaniasis in a VL-endemic area of Nepal, occurring in an immunocompetent patient without prior visceral disease. It emphasizes the importance of considering mucosal leishmaniasis in the differential diagnosis of chronic oral lesions in endemic regions, highlights the role of PCR in confirmation, and supports the use of LAMB in patients who cannot tolerate miltefosine. Recognition of such atypical nondestructive variants broadens our understanding of the clinical and geographic diversity of *L donovani* infection.

## Conflicts of interest

None disclosed.
